# Association between prognosis and SEMA4D/Plexin-B1 expression in various malignancies: A meta-analysis

**DOI:** 10.1097/MD.0000000000013298

**Published:** 2019-02-15

**Authors:** Yibo Yang, Jing Wang, Hui Li, Lihong Liu, Maojin Yao, Tao Xiao

**Affiliations:** a Department of Sport Surgery and Sport Medicine, Hunan Provincial People's Hospital (The First Affiliated Hospital of Hunan Normal University); b Department of Orthopedics, The Second Xiangya Hospital of Central South University, Changsha, Hunan Province, China.; c Department of Microbiology, Immunology, and Cancer Biology, University of Virginia School of Medicine, Charlottesville, VA.

**Keywords:** malignancy, meta-analysis, Plexin-B1, prognosis, SEMA4D

## Abstract

**Introduction:**

SEMA4D and its high affinity receptor Plexin-B1 showed a promising prognosis prediction for carcinoma patients in recent studies, we performed a meta-analysis to evaluate the prognostic role of them in various malignancies.

**Methods:**

A systematic literature search was performed in PubMed, Embase, Web of Science, and CNKI from inception till July 2017. Eligible studies were identified by different reviewers. Hazard ratios (HRs)/related ratios (RRs) and their corresponding 95% confidence intervals (CIs) were extracted to investigate the relevance between malignancies prognosis and SEMA4D/Plexin-B1.

**Results:**

Around 2638 patients from 14 studies were included in this meta-analysis. High expression of SEMA4D was significantly associated with overall survival (OS) and disease-free survival/progression-free survival/recurrence-free survival (DFS/PFS/RFS) in tumors (respectively, HR_os_ = 2.05, 95%CI: 1.68–2.50, *P < *.001; HR_dfs/pfs/rfs_ = 1.59, 95%CI = 1.27–1.98, *P < *.001). However, the relationship between SEMA4D expression and prognosis of breast cancer patients was failed to find (HR = 0.76, 95%CI = 0.32–1.82, *P = *.539). Plexin-B1 level showed a significant positive correlation both with OS and DFS of Caucasian breast cancer patients (respectively, HR_os_ = 0.56, 95%CI: 0.39–0.79, *P = *.001; HR_dfs_ = 0.68, 95%CI = 0.51–0.90, *P = *.008)

**Conclusions:**

SEMA4D could be a prospective biomarker for prognostic prediction of various malignancies except breast cancer. For Caucasian breast cancer patients, SEMA4D's high affinity receptor Plexin-B1 showed a significant positive correlation with survival.

## Abstract 2

Supplemental Digital Content is available in the text

## 1 Introduction

Cancer, one of the greatest worldwide public health issues, remains the leading cause of death in some developing countries.^[1–3]^ Even though a long-term survival rate of breast and colorectal cancer has been increased significantly in most developed countries, other types of cancers and sarcomas are still fatal such as liver and lung cancer.^[3,4]^ Searching tumor biomarkers for diagnosis, treatment, and prognosis is the breaking point of clinical cancer research, such as alpha fetal protein in liver cancer and prostate-specific antigen in prostate cancer.^[5]^

Semaphorin4D (SEMA4D), also known as CD100, is a 150 kD glycoprotein classified as a member of class IV semaphorin family,^[6]^ SEMA4D functions as both a ligand, binding to Plexin-B1 or CD72,^[7]^ and as a receptor.^[8]^ SEMA4D was first found in immune cells, especially highly expressed in resting T cells,^[6]^ and upregulated in B cells, APCs (antigen-presenting cells) when these cells were activated.^[9]^ Initial study suggested that SEMA4D was involved in activation of T cells.^[6]^ SEMA4D enhanced the antibody synthesis of B cells and matured APCs by competitively inhibiting CD72/SHP-1 negative regulation.^[9]^ SEMA4D was observed in embryonic and postnatal CNS (central nervous system), nonspecifically but highly expressing in oligodendrocytes^[10]^ and had been proved as an axonal guidance factor via its high affinity receptor Plexin-B1.^[11]^

Increasing studies of SEMA4D have focused on oncological aspect.^[12–14]^ SEMA4D has a relatively high expression in a series of solid tumor cells comparing with normal tissue cells, such as HNSCC (head and neck squamous cell carcinoma),^[15]^ breast cancer,^[16]^ prostate cancer,^[17]^ CRC (colorectal cancer),^[18,19]^ STS (soft tissue sarcoma),^[13,20]^ EOC (epithelial ovarian cancer),^[21]^ pancreatic cancer,^[22]^ and cervical cancer.^[23]^ Cumulated evidence reveals that SEMA4D participates in tumor angiogenesis,^[12,24]^ regulation of tumor microenvironment^[14]^ and cancer progression.^[25]^ Plexin-B1 that has a high expression on VECs (vascular endothelial cells) surface can activate VECs proangiogenic property after combining with its high affinity ligand SEMA4D.^[12]^ The mechanism how SEMA4D/Plexin-B1 complex promotes angiogenesis is inconclusive, but one thing is confirmed that SEMA4D is an independent angiogenic factor out of other classic molecules such as VEGF-a, bFGF and HGF.^[24]^ SEMA4D also affects tumor microenvironment by negative regulating differentiation of monocytes^[26]^ and TAMs (tumor-associated macrophages) have been proved as a main source of SEMA4D.^[14]^ Moreover, high expressing level of SEMA4D has been proved to predict a worse survival in some carcinomas,^[13,17–23]^ while other study showed a diverse opinion.^[16,27]^ There was no unified conclusion whether SEMA4D can be a promising cancer prognostic biomarker.

Plexin-B1 is a transmembrane receptor which acts through its high affinity ligand SEMA4D, has a series of functions such as regulation of immune cells, axon guidance, tumor angiogenesis, and invasion.^[7]^ Plexin-B1 has an overexpression in colorectal, hepatocellular, breast, pancreatic carcinoma tissue or cell lines.^[19,22,28,29]^ Met or Ron from downstream of SEMA4D/Plexin-B1 is critical for tumor invasive function.^[12]^ Plexin-B1 is also thought to be a predict prognostic marker for several types of tumor, breast cancer in particular.^[16,29–31]^ This meta-analysis is performed to assess the prognostic value of SEMA4D and Plexin-B1 expression in various malignancies.

## 2 Methods

Ethics committee is inapplicable in this meta-analysis.

The present review was conducted according to the guidelines of Preferred Reporting Items for Systematic Reviews and Meta-Analyses (PRISMA)^[32]^ and Meta-Analysis of Observational Studies in Epidemiology group (MOOSE).^[33]^

### 2.1 Search strategy

Literature search was performed in online PubMed (http://www.ncbi.nlm.nih.gov/pubmed), Embase (http://www.embase.com/home), Web of Science (http://wokinfo.com/) and CNKI (http://www.cnki.net/) up to July 1, 2017. Two sets of search terms were adopted for simultaneously retrieval, namely: “CD100 or semaphorin 4D or SEMA4D or Plexin-B1 or semaphorin receptor protein” and “cancer or carcinoma or malignant neoplasm or tumor or benign neoplasm.” No language or other restriction were made. After screening the titles, authors and years, the duplications were removed directly. References from searched publications were manually reviewed for missing relevant literatures. All literatures search was separately performed by 2 reviewers (YYB and LH).

### 2.2 Inclusion and exclusion criteria

The studies were defined eligible in this meta-analysis by following criteria: the patients had been diagnosed any type of cancer or sarcoma, SEMA4D or Plexin-B1 expression was measured from tumor tissues or body fluids, the correlation between SEMA4D or Plexin-B1 expression and patients clinical survival was available, such as either the hazard ratio (HR) or the relative ratio (RR) with corresponding 95% confidence intervals (CIs) or sufficient data which could be used to calculate HRs/RRs and corresponding 95% CIs.

Articles with the following criteria were excluded: case reports, letters, reviews, conference abstracts, and animal or laboratory studies, nondichotomous studies, studies that used the same or overlapped population, studies which were lacked of key data regarding prognosis, study with fewer than 15 patients. Eligible studies were independently and carefully identified from all literatures in triplicate by 2 reviewers (YYB and LH) after discussion.

### 2.3 Data extraction

To rule out any discrepancy, 2 investigators (YYB and LLH) independently evaluated and extracted relevant information according to the guideline of a critical review checklist. The following characteristics were collected from each eligible article, including title, first author's name, year of publication, name of journal, pathological diagnosis of cancer, sample source, origin of population, number of cases, detection method, TNM stage, cut-off value, follow-up, and survival analysis endpoint with corresponding HR/RR and 95% Cls.

The relative ratio (RR) which determined from Cox's multiple regression model was acceptable in this study.^[34]^ HRs/RRs with their 95% CIs were extracted by using the following 2 methods. The univariate analysis results for survival which were reported in eligible studies were considered as the aggregation of the survival data. In most instances, the reported HRs/RRs with corresponding 95% CIs and *P* values were directly derived from the original publications or the E-mails from the authors, with an HR/RR of >1 being associated with elevated risk of mortality or recurrence. Reported HRs/RRs are the most accurate methods. In the absence of HRs/RRs and 95% CIs, the data which were extracted from Kaplan–Meier curves were used to estimate the HRs following the method applied in previous meta-analysis.^[35]^ All the HRs/RRs extraction were performed by all the authors with consensus.

### 2.4 Quality assessment

The quality of eligible study was systematically evaluated according to a critical review checklist of the Dutch Cochrane Centre proposed by MOOSE specifically for prognosis meta-analysis.^[33]^

The Newcastle–Ottawa scale (NOS) for quality of cohort studies was adopted as quality assessment criteria.^[36]^ The evaluated items were classified into 3 aspects including selection of cohorts (4 scores), comparability of cohorts (2 scores) and assessment of outcome (3 scores) with a maximum of 9 scores. High scores evaluation outcome revealed the preciseness of study. The assessments were performed independently by 2 reviewers (YYB and WJ) and aggregated with consensus.

### 2.5 Statistical analysis

All analyses were conducted by mainly using STATA package version 12.0 (STATA Corporation, College Station, TX), and *Z*-test was computed by RevMan version 5.3.5 (Cochrane Collaboration, Oxford, UK).

Pooled HRs with 95% CIs were calculated to evaluated the effect of SEMA4D and Plexin-B1 expression on the survival of cancer. Patients with overexpression of target gene were indicated a poor prognosis if HR > 1 without its 95% CI overlapped with 1. *Z*-test was utilized to evaluate the significance of merged HRs. Heterogeneity of pooled HRs was carried out by using Higgins *I*-square (*I*^2^) and Cochran's *Q*-test statistic. The fixed-effects model (Mantel–Haenszel test) was applied on no significant heterogeneity outcome (*P*_heterogeneity_ > 0.05 or *I*_2_ < 50%).^[37]^ Otherwise, a random-effects model (Der Simonian and Laird method) was used. Subgroup analysis and meta-regression was further performed to explain the source of heterogeneity.^[36,38]^

One-way sensitivity analyses were processed by omitting 1 study at a time to assess the consistency of the combined results. The potential publication bias were assessed by using Begg's funnel plot^[39]^ and Egger's bias.^[40]^ The trim and fill method would be performed if a publication bias existed. All statistical tests were 2-sided, and *P* < .05 was regarded as statistically significant.

## 3 Result

### 3.1 Eligible studies and characteristics

**Figure 1:**
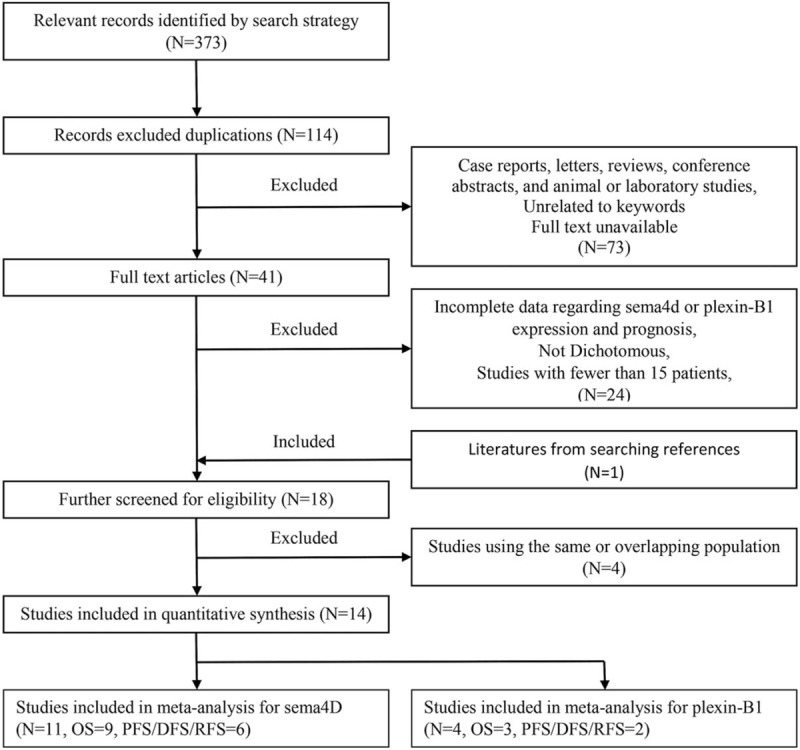
Flow diagram of literatures screening.

As showed in the flow diagram of literatures screening (Fig. 1), a total of 373 articles were originally searched from PubMed, Embase, Web of Science, and CNKI. Full text screening was performed based on the inclusion and exclusion criteria, and 18 candidate studies were eligible. When data extraction due to using overlapping cohort 4 literatures was further excluded. Finally, 14 articles were qualified for our meta-analysis,^[13,16–23,29–31,41,42]^ 11 for SEMA4D^[13,16–23,41,42]^ and 4 ^[16,29–31]^ for its receptor Plexin-B1. Of the SEMA4D related studies, 9 for overall survival (OS), 6 for disease-free survival (DFS)/progression-free survival (PFS)/recurrence-free survival (RFS). Of the Plexin-B1 related studies, 3 for OS and 2 for DFS.

The requisite data was extracted from 14 eligible studies and integrated into Table 1. A total of 1375 patients from United States, China, Brazil, Japan, and Pakistan were included in SEMA4D group while 1410 patients from Pakistan, Germany and Netherlands were included in Plexin-B1 group. Interestingly, all 4 articles of Plexin-B1 group focused on breast cancer research, and SEMA4D group showed a wide variety of malignant tumors including prostate cancer, colorectal cancer (CRC), soft tissue sarcoma (STS), epithelial ovarian cancer (EOC), breast cancer, cervical cancer, and pancreatic cancer. The commonest method to detect SEMA4D expression in selected studies was immunohistochemistry (IHC) staining, while the majority of studies evaluated Plexin-B1 expression by microarray. Staining assessment score was used to set up the dichotomous cut-off value in all IHC studies. The rest of literatures mostly used Median as cut-off value. There were 13 studies used tumor tissue as sample, within them there was one study took ascites as comparison to tissue, besides one study used blood.

**Table 1:**
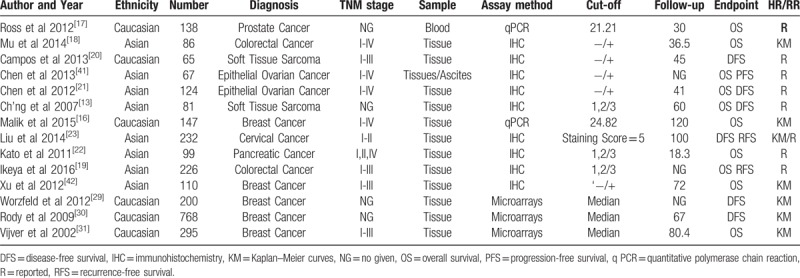
The requisite characteristic of all 14 eligible studies.

The Newcastle–Ottawa scale (NOS) was used to assess the methodological quality of eligible literatures. All papers’ NOS ranged from 5 to 9 (Table 2), with an average of 7.12. Study with scores≥7 was defined a high-quality record, otherwise low.

**Table 2:**
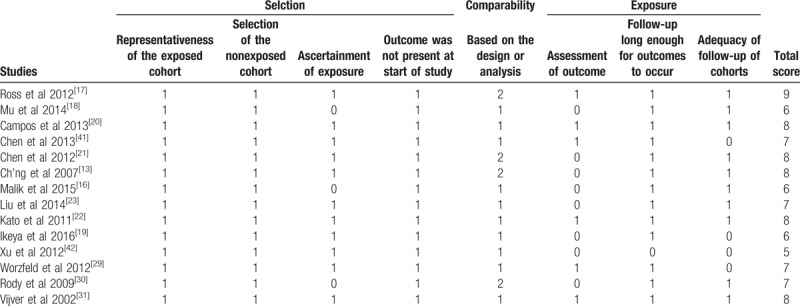
The Newcastle–Ottawa scale for methodological quality of eligible literatures.

### 3.2 Evidence synthesis

**Figure 2:**
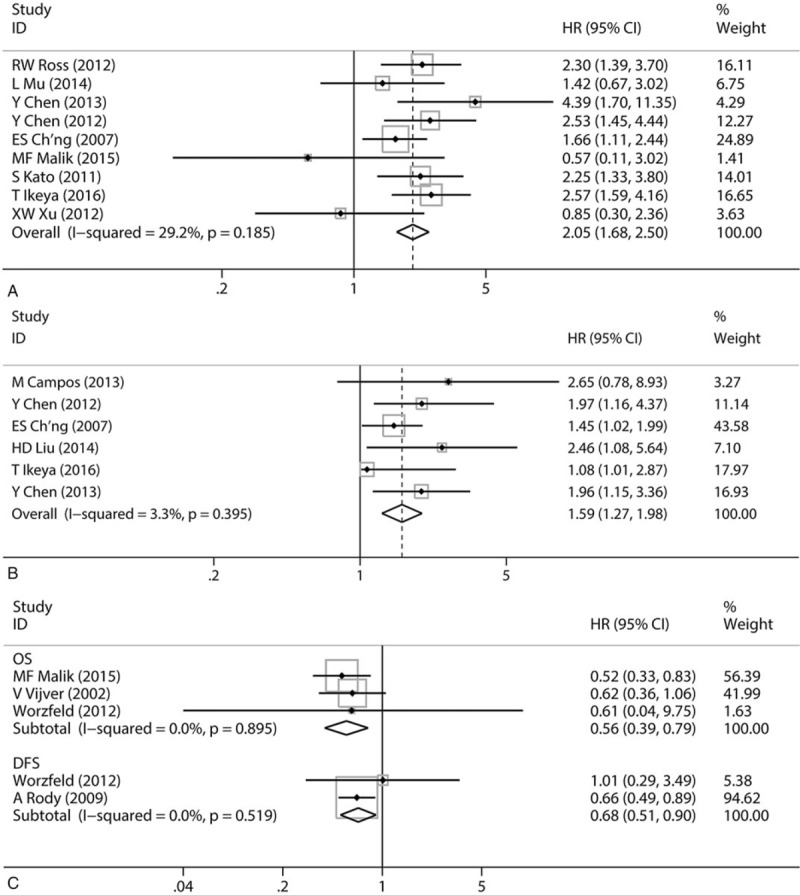
(A) Forest plot illustrating correlation between Sema4d expression and overall survival in various malignancies. (B) Forest plot illustrating correlation between Sema4d expression and DFS/PFS/RFS in various malignancies. (C) Forest plot illustrating correlation between Plexin-B1 expression and survivals in various malignancies.

All pooled HRs and heterogeneity results are shown in Table 3 (Supplemental figure 1–8) which is divided into SEMA4D sublist and Plexin-B1 sublist. A fixed effect model was applied to pool HRs/RRs from 9 SEMA4D OS studies included 1078 patients. The combined HR is 2.05 with corresponding 95%Cl 1.68–2.50, which revealed that overexpression of SEMA4D may predict a poor prognosis of multiple malignancies (Fig. 2A). Subgroup analyses of overall survival were categorized by ethnicity of patients, diagnosis of diseases, assay method, sample, source of HRs/RRs and quality assessed classification. There were significant associations between high level expression of SEMA4D and poor survival in Asian patients (HR = 2.05, 95%Cl: 1.65–2.54, *P < *.001), in patients with colorectal cancer (HR = 2.16, 95%Cl: 1.44–3.25, *P < *.001) and epithelial ovarian cancer (HR = 2.92, 95%CI: 1.80–4.73, *P < *.001), in studies which examined SEMA4D with immunohistochemistry staining (HR = 2.05, 95%Cl:1.65–2.54, *P < *.001), in tissue samples (HR = 2.01, 95%CI: 1.62–2.49, *P < *.001), in studies which reported HRs/RRs (HR = 2.23, 95%CI: 1.81–2.75, *P < *.001) and in high methodological quality studies (HR = 2.07, 95%CI: 1.63–2.62, *P < *.001) (Supplemental figures 1–5).

**Table 3:**
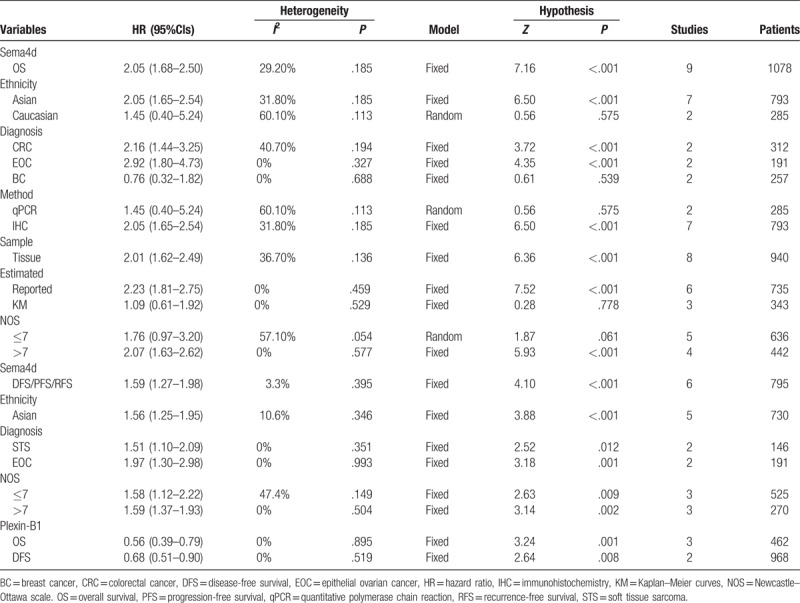
All pooled HRs and heterogeneity results.

Similar to total OS analysis result, the high expression of SEMA4D was significantly associated with a poor DFS/PFS/RFS basing on a fixed effects model analysis HRs/RRs from 6 studies with 795 patients (HR = 1.59, 95%CI: 1.27–1.98, *P < *.001) (Fig. 2B). Subgroup analyses of DFS/PFS/RFS were performed in the same categories. Correlations between SEMA4D level and DFS/PFS/RFS were observed in Asian group (HR = 1.56, 95%CI: 1.25–1.95, *P < *.001), in soft tissue sarcoma group (HR = 1.51, 95%CI: 1.10–2.09, *P = *.012) and epithelial ovarian cancer group (HR = 1.97, 95%CI: 1.30–2.98, *P = *.001), in both high and low methodological quality groups (respectively, HR_Hi_ = 1.59, 95%CI: 1.37–1.93, *P < *.001; HR_Lo_ = 1.76, 95%Cl: 1.12–2.22, *P < *.001). Because all 6 studies assessed SEMA4D expression from tumor tissue by immunohistochemistry staining and reported the HRs/RRs, IHC group, tissue group and reported HRs/RRs group had a same result as the total DFS/PFS/RFS analysis (HR = 1.59, 95%CI: 1.27–1.98, *P < *.001) (Supplemental figures 6–8).

As noted, all 4 articles of Plexin-B1 group focused on Caucasian breast cancer research, so we only evaluated the relationship between Plexin-B1 level and Caucasian breast cancer patients’ survival. It turned out that elevated Plexin-B1 showed a significant association with favorable OS (HR = 0.56, 95%CI: 0.39–0.79, *P = *.001) and DFS (HR = 0.68, 95%CI: 0.51–0.90, *P = *.008) (Fig. 2C).

### 3.3 Heterogeneity analysis

All nonsubgroup pooled HRs were calculated in fixed effect model because of their low or no heterogeneity. Precisely, heterogeneity was found among the SEMA4D OS studies (*P*_heterogeneity_ = 0.185, *I*^2^ = 29.2%). To investigate the source of heterogeneity in SEMA4D OS group, a meta-regression was utilized to assess by year of publication, quality classification, sample, ethnicity, assay method and diagnosis, such as breast cancer, colorectal cancer and epithelial ovarian cancer. All main results were shown in Table 4. Breast cancer category (*P* = 0.057) dominantly induced heterogeneity rather than others categories.

**Table 4:**
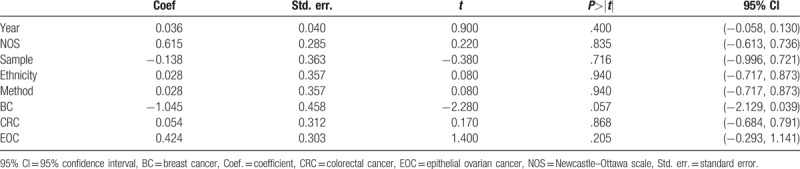
The meta regression result of the SEMA4D overall survival studies showed heterogeneity mainly came from breast cancer studies group.

### 3.4 Publication bias and sensitivity analysis

**Figure 3:**
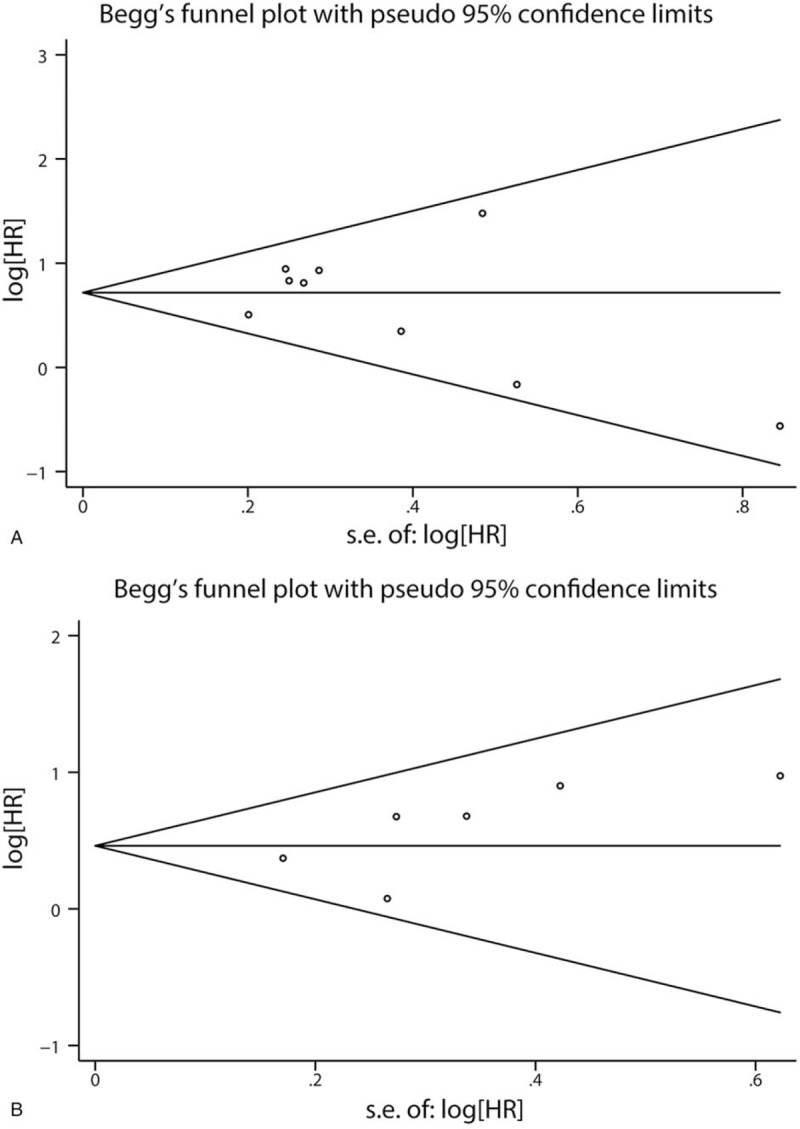
(A) Begg's funnel plot for publication bias of 9 studies in Sema4D OS group. (B) Begg's funnel plot for publication bias of 6 studies in Sema4D DFS/PFS/RFS group.

Publication bias was evaluated by Begg's funnel plot and Egger's test in OS group with 9 literatures and DFS/PFS/RFS group with 6 studies for SEMA4D. The Egger's test outcome (Table 5, *P* > .05) and symmetrical Begg's funnel plots (Fig. 3) showed no potential publication bias.

**Table 5:**
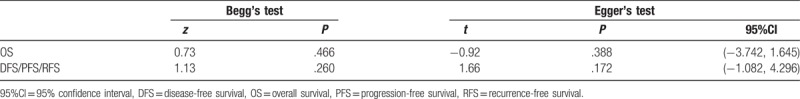
Begg's test and Egger's test for publication bias analysis on SEMA4D studies.

**Figure 4:**
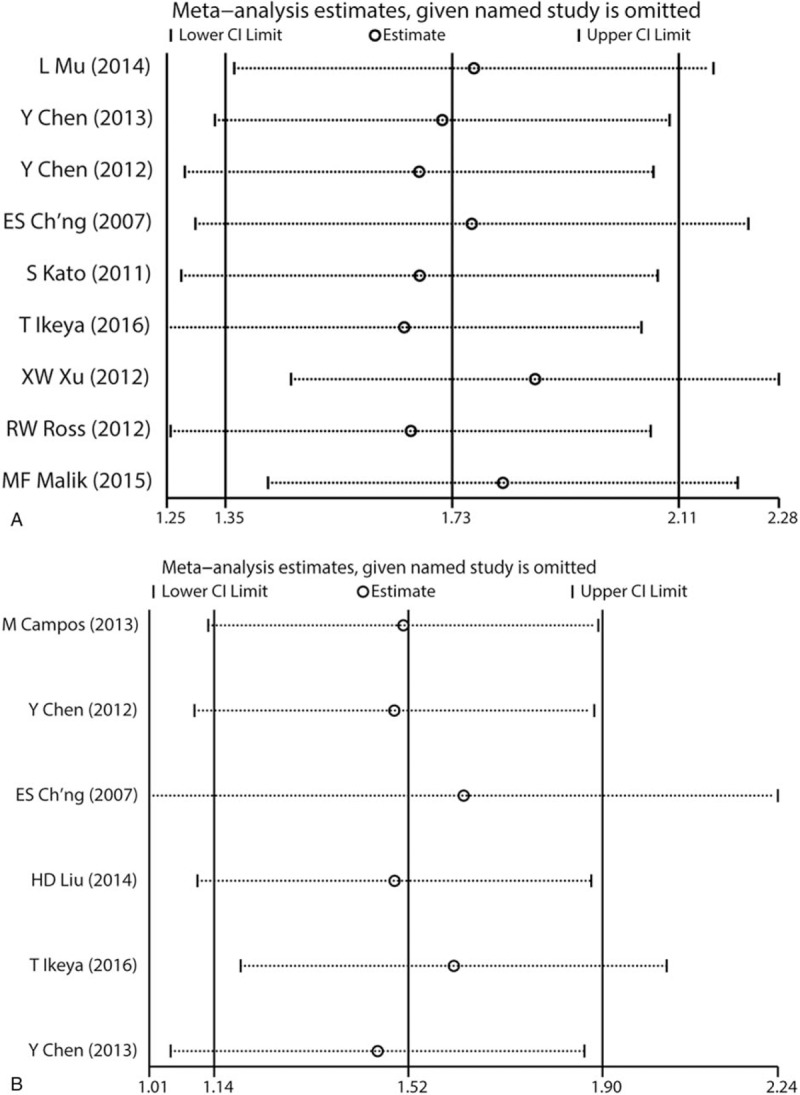
(A) Sensitivity analyses of SEMA4D OS group. (B) Sensitivity analyses of SEMA4D DFS/PFS/RFS group.

Sensitivity analyses of SEMA4D OS group and DFS/PFS/RFS group showed no significant variation on original result by omitting individual study (Fig. 4).

The publication bias and sensitivity analyses result of Plexin-B1 were insignificant base on there were only 3 studies in OS group and 2 in DFS group.

## 4 Discussion

Neoplastic biomarkers are essential tools for cancer diagnosis, clinical therapy and survival. An ideal prognostic biomarker should match 2 features: accurate measurability and definite association with either pathologic progression or prognosis.^[43]^ SEMA4D are widely utilized in clinical pathology examination to auxiliary diagnose neurogenic tumor.^[44]^ Substantial evidence demonstrated that SEMA4D was involved in angiogenesis, regulation of tumor microenvironment and cancer progression of various types of tumors. Moreover, an anti-SEMA4D monoclonal antibody named VX15/2503 has been proved its broad-spectrum anti-tumor property in mouse and is entering clinical trials.^[45]^ However, no evidence proves novel insight that SEMA4D can be utilized as a prognostic biomarker in diverse malignant tumors.

In our cognizance, the present analysis is the first study to identify the prognosis predictive potency of SEMA4D in various types of malignancies. By collecting and combining survival indexes from all eligible literatures, we concluded that rising expression of SEMA4D was significantly associated with OS and DFS/PFS/RFS in tumors (respectively, HR_os_ = 2.05, 95%CI: 1.68–2.50, *P < *.001; HR_dfs/pfs/rfs_ = 1.59, 95%CI = 1.27–1.98, *P < *.001). Heterogeneity which existed in overall survival analyses (*I*^2^ = 29.3%, *P = *.185) was mainly attributed to the outcome of breast cancer patients. Besides, the relationship between SEMA4D expression and prognosis of breast cancer patients was failed to find (HR = 0.76, 95%CI = 0.32–1.82, *P = *.539). These statistical negative results revealed that SEMA4D probably was not an accurate prognosis predictor in breast cancer. The pooled survival results of Plexin-B1, SEMA4D high affinity receptor, precisely remedied the insufficient in breast cancer studies: Plexin-B1 level showed a significant positive correlation both with OS and DFS (respectively, HR_os_ = 0.56, 95%CI: 0.39–0.79, *P = *.001; HR_dfs_ = 0.68, 95%CI = 0.51–0.90, *P = *.008).

SEMA4D is over-expressed in various tumor tissues.^[46]^ Abundant vivo evidence revealed SEMA4D effected tumor progress by regulating tumor angiogenesis and tumor environment.^[14,41]^ Most of selected studies support a consensus that SEMA4D acts as an angiogenic promoter mainly through Plexin-B1 rather than VEFG-a.^[13,16–23]^ Base on the over-expression of Plexin-B1 in endothelial cells, SEMA4D/Plexin-B1 signal efficiently enhances endothelium migration.^[24]^ The mechanism of SEMA4D tubulogenesis is mainly because of the activation of tyrosine kinase Met and Rho pathways.^[12,24]^ Interestingly, grafting SEMA4D over-expressing melanoma to Plexin-B1 deficient mouse shows no significant reduction of neovascularization.^[28]^ It is probably because that Plexin-B1 is not the essential for SEMA4D promoting tumor angiogenesis, or maybe because SEMA4D/Plexin-B1 interaction only functions at the early stage of tumor formation.

Moreover, SEMA4D regulates tumor environment by inhibiting monocyte migration and prompting monocyte differentiation to M2 macrophages which acts as a tumor-promotor.^[41]^ Chen's study^[41]^ showed a strong association between SEMA4D expression and M2 macrophages count both in epithelial ovarian cancer (EOC) tumor sample and in malignant ascites.

SEMA4D does not seem to be an accurate prognosis predictor for breast cancer base on the result of our study (HR = 0.76, 95%CI = 0.32–1.82, *P = *.539). Fortunately, there were several literatures^[29–31,42]^ which demonstrated the inverse correlation between Plexin-B1 and prognosis of patients. Swiercz's study^[47]^ indicated Plexin-B1 stimulated tumor cell migration via tyrosine kinase receptor ErbB-2 pathway while antimigrated via met pathway, He believed that the effect of SEMA4D/Plexin-B1 mainly depended on the superior pathway. Based on our result, regardless of ErbB-2 or Met took charge, Plexin-B1 was an independent prognosis marker for breast cancer patients.

Nevertheless, there are several limitations of the present analysis. First, the number of the cancer types which included in this meta-analysis were inadequate. There were 7 kinds of cancer in 14 studies, including 1520 breast cancer patients and only 99 pancreatic cancer patients. The small sample size can unavoidably cause sample bias and random errors. Thus, more studies with larger population are necessary for further analysis. Second, several HRs were calculated based on Kaplan–Meier curves; some minor errors were generated during calculation. Third, the cut-off value of SEMA4D and Plexin-B1 expression were various. Besides, IHC staining assessment which were used to set up the dichotomous cut-off value was lack of unified and objective criterion for staining evaluation.^[48]^ Fourth, all Plexin-B1 group articles were breast cancer researches and all these researches were focus on Caucasian. As we all known, Caucasian has significantly higher incidence and mortality rates of breast cancer than Asian and African,^[49]^ and there was no evidence proved that Plexin-B1 expression had a relationship with the survival of Asian and African breast cancer patients. Finally, all eligible studies in our meta-analysis were published in English and Chinese, in spite of no language restriction in Search Strategy that will cause language bias.

## 5 Conclusion

In summary, the present analysis demonstrated that SEMA4D could be a prospective biomarker for prognostic prediction of various malignancies except breast cancer. For Caucasian breast cancer patients, SEMA4D's high affinity receptor Plexin-B1 showed a significant positive correlation with survival. However, the eligible studies are insufficient, more comprehensive studies are needed to support this conclusion.

## Author contributions

**Conceptualization:** Yibo Yang.

**Data curation:** Yibo Yang, Hui Li.

**Formal analysis:** Yibo Yang.

**Funding acquisition:** Hui Li, Tao Xiao.

**Methodology:** Yibo Yang, Hui Li.

**Project administration:** Yibo Yang, Jing Wang, Lihong Liu.

**Resources:** Yibo Yang.

**Software:** Yibo Yang.

**Supervision:** Maojin Yao.

**Validation:** Yibo Yang.

**Writing – original draft:** Yibo Yang.

**Writing – review & editing:** Yibo Yang, Jing Wang, Maojin Yao, Tao Xiao.

## Supplementary Material

Supplemental Digital Content

## References

[1] Bray F Ferlay J Soerjomataram I Global cancer statistics 2018: GLOBOCAN estimates of incidence and mortality worldwide for 36 cancers in 185 countries. CA Cancer J Clin 2018;68:394–424. Epub ahead of print. 30207593 10.3322/caac.21492

[2] Siegel RL Miller KD Jemal A Cancer statistics, 2018. CA Cancer J Clin 2018;68:7–30. 29313949 10.3322/caac.21442

[3] Chen W Zheng R Baade PD Cancer statistics in China, 2015. CA Cancer J Clin 2016;66:115–32. 26808342 10.3322/caac.21338

[4] Liu L Global sex differences in cancer mortality with age and country specific characteristics. Asian Pac J Cancer Prev 2016;17:3469–76. 27509994

[5] Song P Tang Q Feng X Biomarkers: evaluation of clinical utility in surveillance and early diagnosis for hepatocellular carcinoma. Scand J Clin Lab Invest Suppl 2016;245:S70–76. 27438343 10.1080/00365513.2016.1210328

[6] Bougeret C Mansur IG Dastot H Increased surface expression of a newly identified 150-kDa dimer early after human T lymphocyte activation. J Immunol 1992;148:318–23. 1530858

[7] Tamagnone L Artigiani S Chen H Plexins are a large family of receptors for transmembrane, secreted, and GPI-anchored semaphorins in vertebrates. Cell 1999;99:71–80. 10520995 10.1016/s0092-8674(00)80063-x

[8] Herold C Bismuth G Bensussan A Activation signals are delivered through two distinct epitopes of CD100, a unique 150 kDa human lymphocyte surface structure previously defined by BB18 mAb. Int Immunol 1995;7:1–8. 7718506 10.1093/intimm/7.1.1

[9] Hall KT Boumsell L Schultze JL Human CD100, a novel leukocyte semaphorin that promotes B-cell aggregation and differentiation. Proc Natl Acad Sci U S A 1996;93:11780–5. 8876214 10.1073/pnas.93.21.11780 PMC38135

[10] Moreau-Fauvarque C Kumanogoh A Camand E The transmembrane semaphorin Sema4D/CD100, an inhibitor of axonal growth, is expressed on oligodendrocytes and upregulated after CNS lesion. J Neurosci 2003;23:9229–39. 14534257 10.1523/JNEUROSCI.23-27-09229.2003 PMC6740837

[11] Masuda K Furuyama T Takahara M Sema4D stimulates axonal outgrowth of embryonic DRG sensory neurones. Genes Cells 2004;9:821–9. 15330859 10.1111/j.1365-2443.2004.00766.x

[12] Conrotto P Valdembri D Corso S Sema4D induces angiogenesis through Met recruitment by Plexin B1. Blood 2005;105:4321–9. 15632204 10.1182/blood-2004-07-2885

[13] Ch’ng E Tomita Y Zhang B Prognostic significance of CD100 expression in soft tissue sarcoma. Cancer 2007;110:164–72. 17520683 10.1002/cncr.22764

[14] Sierra JR Corso S Caione L Tumor angiogenesis and progression are enhanced by Sema4D produced by tumor-associated macrophages. J Exp Med 2008;205:1673–85. 18559453 10.1084/jem.20072602 PMC2442644

[15] Takada H Ibaragi S Eguchi T Semaphorin 4D promotes bone invasion in head and neck squamous cell carcinoma. Int J Oncol 2017;51:625–32. 28656278 10.3892/ijo.2017.4050 PMC8353226

[16] Malik MF Ye L Jiang WG Reduced expression of semaphorin 4D and plexin-B in breast cancer is associated with poorer prognosis and the potential linkage with oestrogen receptor. Oncol Rep 2015;34:1049–57. 26035216 10.3892/or.2015.4015

[17] Ross RW Galsky MD Scher HI A whole-blood RNA transcript-based prognostic model in men with castration-resistant prostate cancer: a prospective study. Lancet Oncol 2012;13:1105–13. 23059047 10.1016/S1470-2045(12)70263-2

[18] Mu L Wang J Guo X [Correlation and clinical significance of expressions of HIF-1alpha and Sema4D in colorectal carcinoma tissues]. Zhonghua Wei Chang Wai Ke Za Zhi 2014;17:388–92. 24760652

[19] Ikeya T Maeda K Nagahara H The combined expression of Semaphorin4D and PlexinB1 predicts disease recurrence in colorectal cancer. BMC Cancer 2016;16:525. 27456345 10.1186/s12885-016-2577-6 PMC4960918

[20] Campos M SG DEC Ribeiro GG Ki-67 and CD100 immunohistochemical expression is associated with local recurrence and poor prognosis in soft tissue sarcomas, respectively. Oncol Lett 2013;5:1527–35. 23759874 10.3892/ol.2013.1226 PMC3678859

[21] Chen Y Zhang L Pan Y Over-expression of semaphorin4D, hypoxia-inducible factor-1alpha and vascular endothelial growth factor is related to poor prognosis in ovarian epithelial cancer. Int J Mol Sci 2012;13:13264–74. 23202951 10.3390/ijms131013264 PMC3497325

[22] Kato S Kubota K Shimamura T Semaphorin 4D, a lymphocyte semaphorin, enhances tumor cell motility through binding its receptor, plexinB1, in pancreatic cancer. Cancer Sci 2011;102:2029–37. 21812859 10.1111/j.1349-7006.2011.02053.x

[23] Liu H Yang Y Xiao J Semaphorin 4D expression is associated with a poor clinical outcome in cervical cancer patients. Microvasc Res 2014;93:1–8. 24603190 10.1016/j.mvr.2014.02.007

[24] Basile JR Barac A Zhu T Class IV semaphorins promote angiogenesis by stimulating Rho-initiated pathways through plexin-B. Cancer Res 2004;64:5212–24. 15289326 10.1158/0008-5472.CAN-04-0126

[25] Trusolino L Comoglio PM Scatter-factor and semaphorin receptors: cell signalling for invasive growth. Nat Rev Cancer 2002;2:289–300. 12001990 10.1038/nrc779

[26] Delaire S Billard C Tordjman R Biological activity of soluble CD100. II. Soluble CD100, similarly to H-SemaIII, inhibits immune cell migration. J Immunol 2001;166:4348–54. 11254688 10.4049/jimmunol.166.7.4348

[27] Rody A Holtrich U Gaetje R Poor outcome in estrogen receptor-positive breast cancers predicted by loss of plexin B1. Clin Cancer Res 2007;13:1115–22. 17317819 10.1158/1078-0432.CCR-06-2433

[28] Fazzari P Penachioni J Gianola S Plexin-B1 plays a redundant role during mouse development and in tumour angiogenesis. BMC Dev Biol 2007;7:55. 17519029 10.1186/1471-213X-7-55 PMC1890291

[29] Worzfeld T Swiercz JM Looso M ErbB-2 signals through Plexin-B1 to promote breast cancer metastasis. J Clin Invest 2012;122:1296–305. 22378040 10.1172/JCI60568 PMC3314465

[30] Rody A Karn T Ruckhaberle E Loss of Plexin B1 is highly prognostic in low proliferating ER positive breast cancers--results of a large scale microarray analysis. Eur J Cancer 2009;45:405–13. 10.1016/j.ejca.2008.10.016 19054665

[31] van de Vijver MJ He YD van’t Veer LJ A gene-expression signature as a predictor of survival in breast cancer. N Engl J Med 2002;347:1999–2009. 12490681 10.1056/NEJMoa021967

[32] Moher D Liberati A Tetzlaff J Preferred reporting items for systematic reviews and meta-analyses: the PRISMA statement. J Clin Epidemiol 2009;62:1006–12. 19631508 10.1016/j.jclinepi.2009.06.005

[33] Stroup DF Berlin JA Morton SC Meta-analysis of observational studies in epidemiology: a proposal for reporting. Meta-analysis Of Observational Studies in Epidemiology (MOOSE) group. JAMA 2000;283:2008–12. 10789670 10.1001/jama.283.15.2008

[34] Ouyang Y Li H Bu J Hypoxia-inducible factor-1 expression predicts osteosarcoma patients’ survival: a meta-analysis. Int J Biol Markers 2016;31:e229–234. 27312586 10.5301/jbm.5000216

[35] Tierney JF Stewart LA Ghersi D Practical methods for incorporating summary time-to-event data into meta-analysis. Trials 2007;8:16. 17555582 10.1186/1745-6215-8-16 PMC1920534

[36] Stang A Critical evaluation of the Newcastle–Ottawa scale for the assessment of the quality of nonrandomized studies in meta-analyses. Eur J Epidemiol 2010;25:603–5. 20652370 10.1007/s10654-010-9491-z

[37] Mantel N Haenszel W Statistical aspects of the analysis of data from retrospective studies of disease. J Natl Cancer Inst 1959;22:719–48. 13655060

[38] Thompson SG Higgins JP How should meta-regression analyses be undertaken and interpreted? Stat Med 2002;21:1559–73. 12111920 10.1002/sim.1187

[39] Begg CB Mazumdar M Operating characteristics of a rank correlation test for publication bias. Biometrics 1994;50:1088–101. 7786990

[40] Egger M Davey Smith G Schneider M Bias in meta-analysis detected by a simple, graphical test. BMJ 1997;315:629–34. 9310563 10.1136/bmj.315.7109.629 PMC2127453

[41] Chen Y Zhang L Lv R Overexpression of Semaphorin4D indicates poor prognosis and prompts monocyte differentiation toward M2 macrophages in epithelial ovarian cancer. Asian Pac J Cancer Prev 2013;14:5883–90. 24289594 10.7314/apjcp.2013.14.10.5883

[42] Xu X The expressions of Sema4D and HER-2 proteins in breast cancers and their clinical significance. J Chin Phys 2012;14:1017–21.

[43] Tormey DC Waalkes TP Biological markers as prognostic and clinical evaluation tools. Eur J Cancer 1980;suppl 1:21–4. 7032933

[44] Rizzolio S Tamagnone L Semaphorin signals on the road to cancer invasion and metastasis. Cell Adh Migr 2007;1:62–8. 19329883 10.4161/cam.1.2.4570 PMC2633973

[45] Fisher TL Seils J Reilly C Saturation monitoring of VX15/2503, a novel semaphorin 4D-specific antibody, in clinical trials. Cytometry B Clin Cytom 2016;90:199–208. 26566052 10.1002/cyto.b.21338 PMC5064733

[46] Ch’ng ES Kumanogoh A Roles of Sema4D and Plexin-B1 in tumor progression. Mol Cancer 2010;9:251. 20858260 10.1186/1476-4598-9-251 PMC2955613

[47] Swiercz JM Worzfeld T Offermanns S ErbB-2 and met reciprocally regulate cellular signaling via plexin-B1. J Biol Chem 2008;283:1893–901. 18025083 10.1074/jbc.M706822200

[48] Varghese F Bukhari AB Malhotra R IHC Profiler: an open source plugin for the quantitative evaluation and automated scoring of immunohistochemistry images of human tissue samples. PLoS One 2014;9:e96801. 24802416 10.1371/journal.pone.0096801 PMC4011881

[49] DeSantis CE Fedewa SA Goding Sauer A Breast cancer statistics, 2015: Convergence of incidence rates between black and white women. CA Cancer J Clin 2016;66:31–42. 26513636 10.3322/caac.21320

