# Supplementary material for: Association between prognosis and SEMA4D/Plexin-B1 expression in various malignancies: A meta-analysis
Source: Medicine (Baltimore). 2019 Feb 15;98(7):e13298. doi: 10.1097/MD.0000000000013298 (PMC6407964; doi:10.1097/MD.0000000000013298)

Supplemental figure 1 Subgroup analyses forest plot illustrating correlation between Sema4d expression and overall survival in various malignancies, categorized by diagnosis.


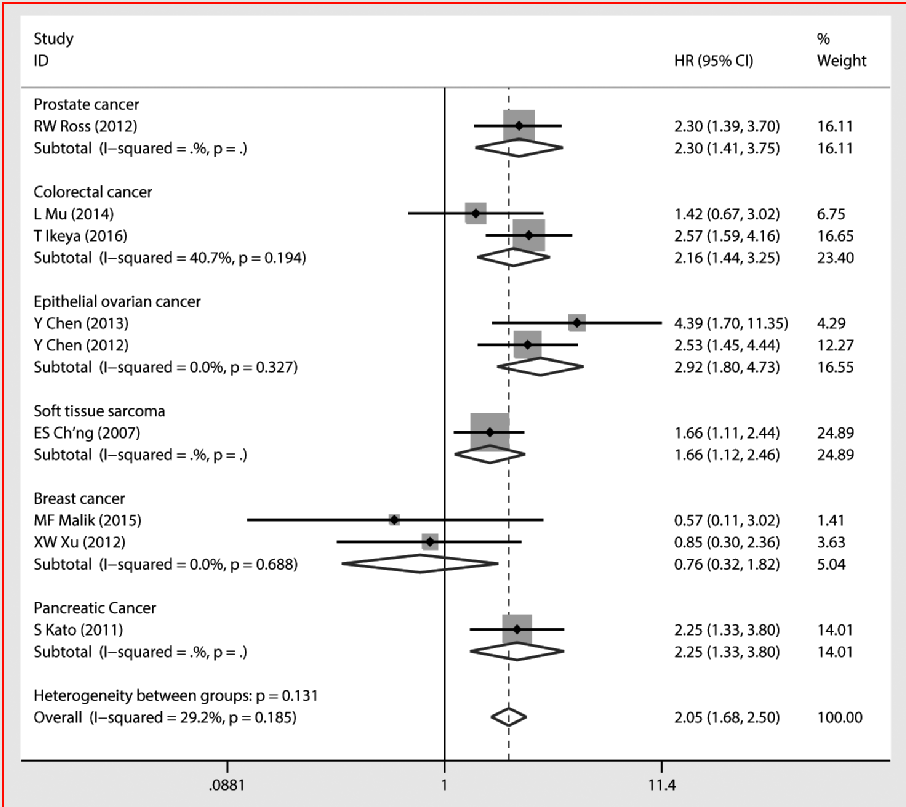


Supplemental figure 2 Subgroup analyses forest plot illustrating correlation between Sema4d expression and overall survival in various malignancies, categorized by ethnicity/method.


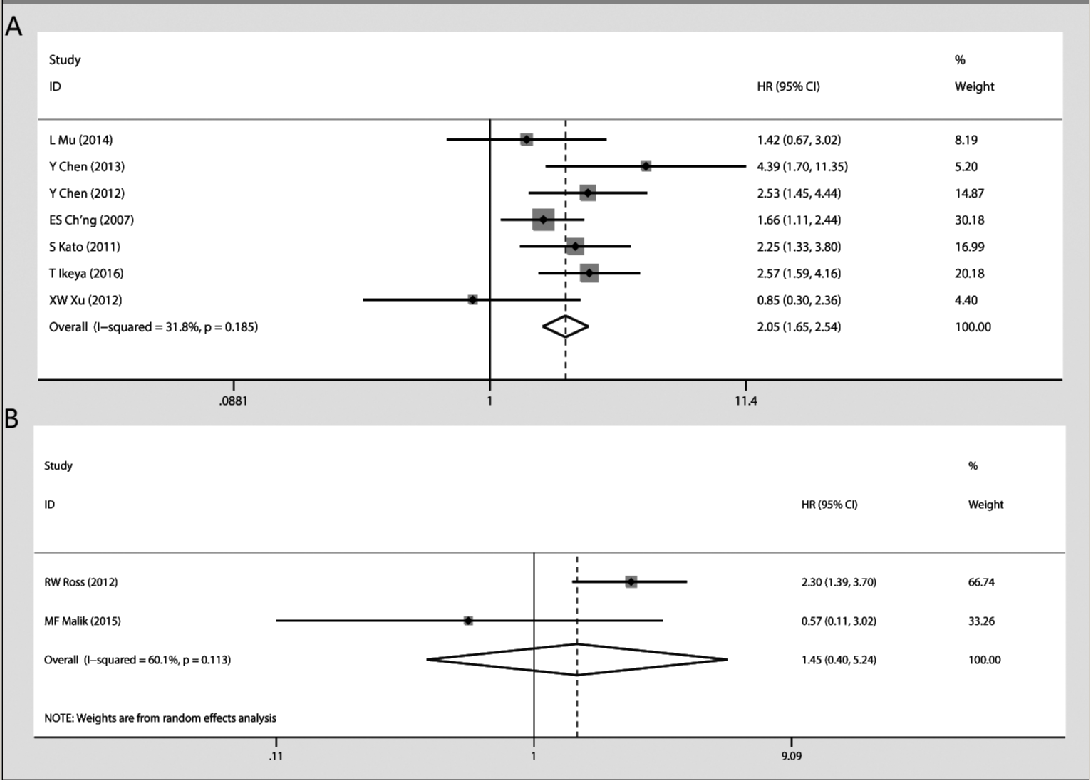


Supplemental figure 2A shows Asian patients’ overall survival correlated to Sema4d expression (also as the data measured by IHC).

Supplemental figure 2B shows Caucasian patients’ overall survival correlated to Sema4d expression(also as the data measured by qPCR).

Supplemental figure 3 Subgroup analyses forest plot illustrating correlation between Sema4d expression and overall survival in various malignancies, categorized by sample.


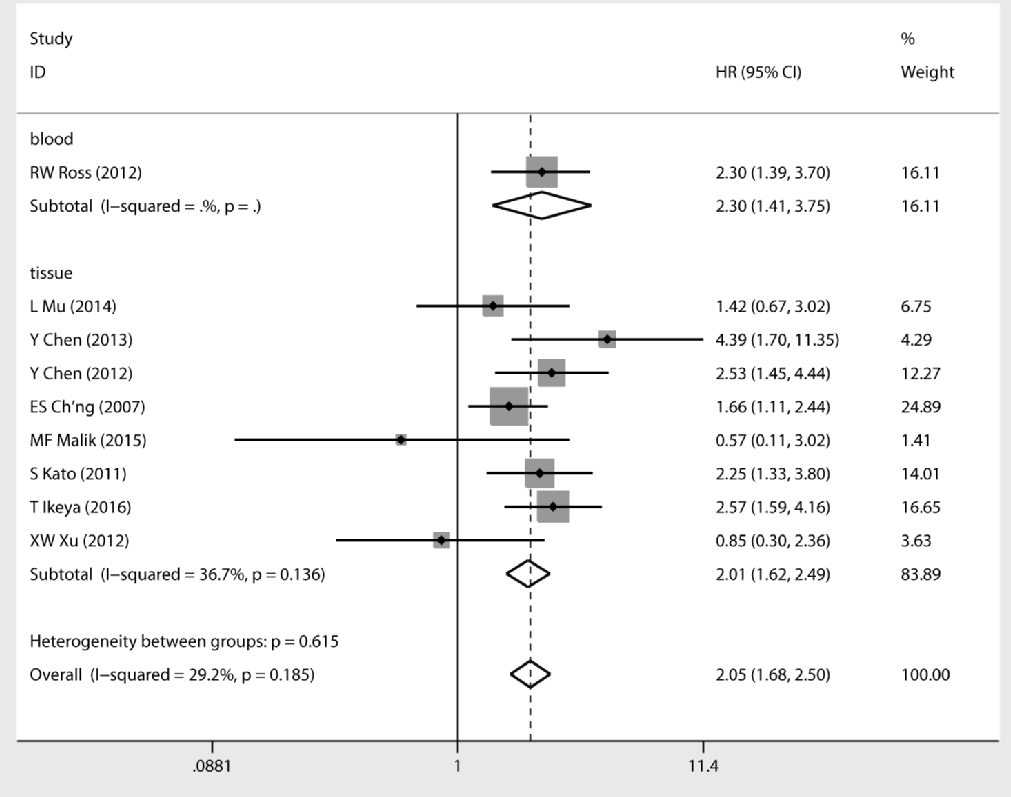


Supplemental figure 4 Subgroup analyses forest plot illustrating correlation between Sema4d expression and overall survival in various malignancies, categorized by HR source. R: reported; KM: Kaplan-Meier curves.


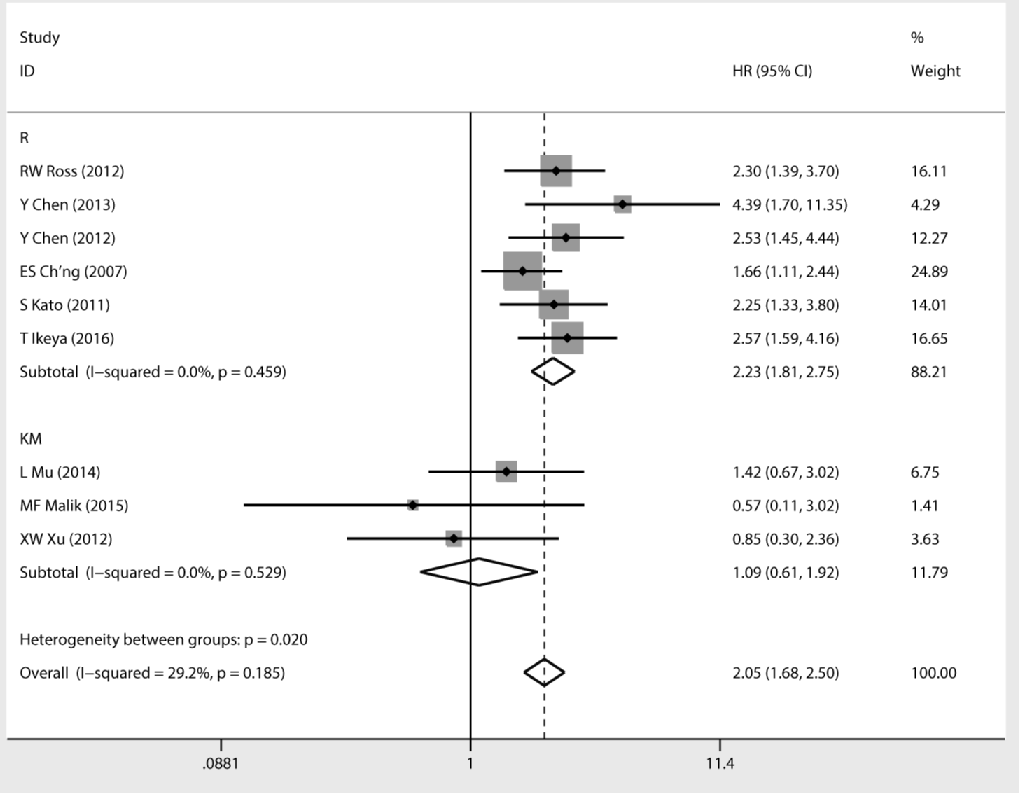


Supplemental figure 5 Subgroup analyses forest plot illustrating correlation between Sema4d expression and overall survival in various malignancies, categorized by NOS.


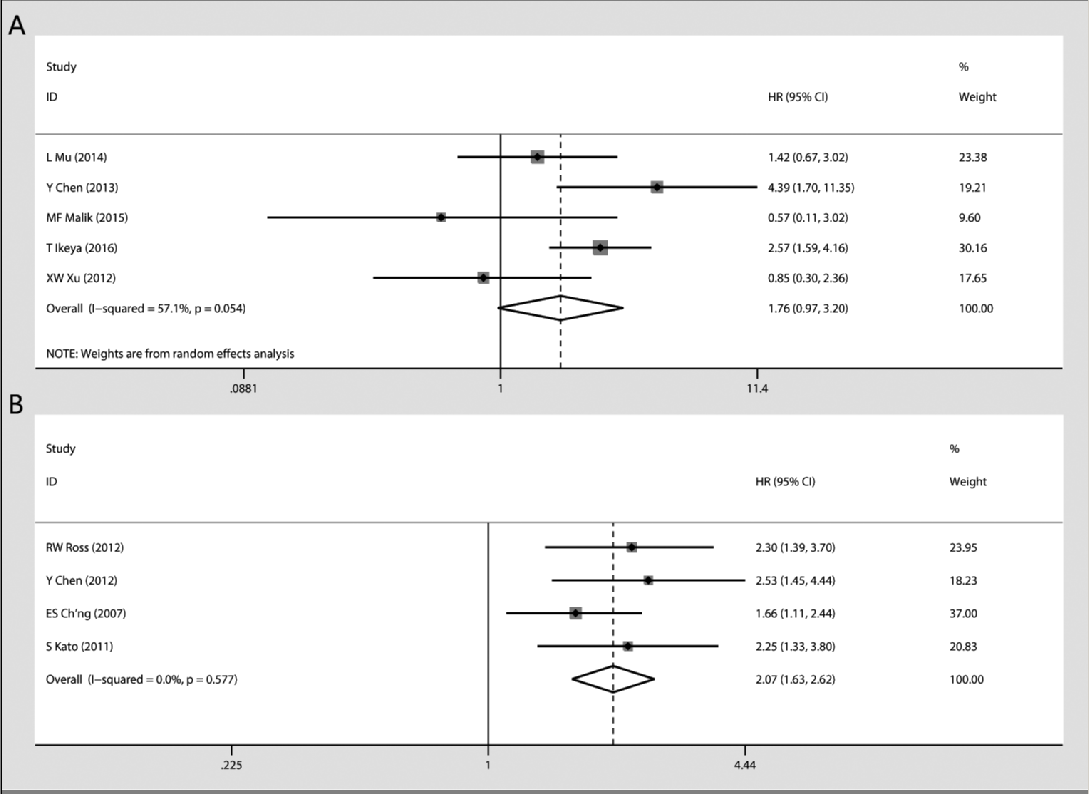


Supplemental figure 5A shows result from researches which got NOS ≤7.

Supplemental figure 5B shows result from researches which got NOS ＞7.

Supplemental figure 6 Subgroup analyses forest plot illustrating correlation between Sema4d expression and DFS/PFS/RFS in various malignancies, categorized by ethnicity.


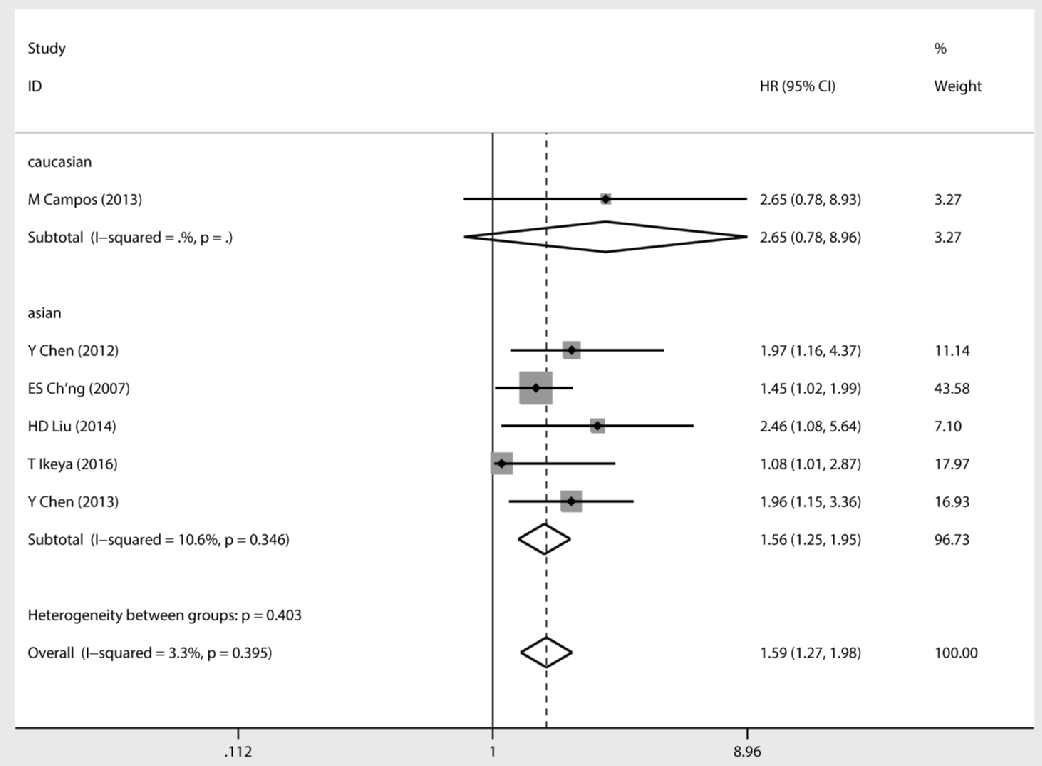


Supplemental figure 7 Subgroup analyses forest plot illustrating correlation between Sema4d expression and DFS/PFS/RFS in various malignancies, categorized by diagnosis.


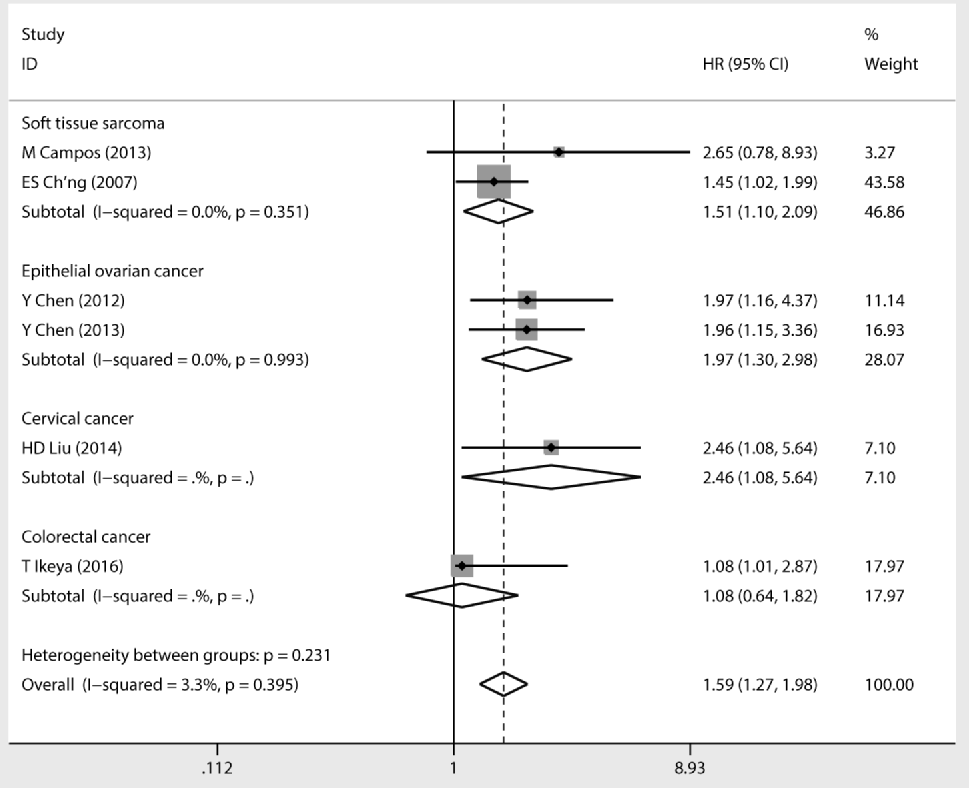


Supplemental figure 8 Subgroup analyses forest plot illustrating correlation between Sema4d expression and DFS/PFS/RFS in various malignancies, categorized by NOS.


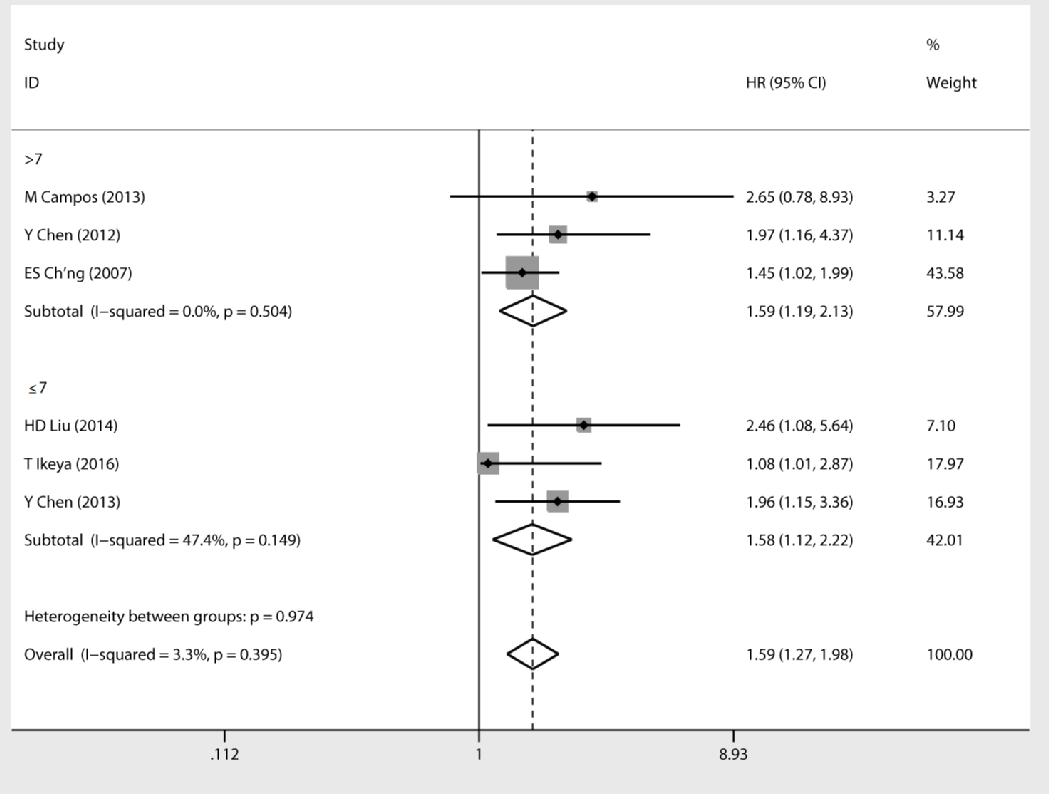

Supplement: Supplemental Digital Content [file medi-98-e13298-s001.doc]
